# Positive Impact of Holistic Nursing on Cognitive Impairment and Psychiatric Symptoms in Patients With Alzheimer’s Disease

**DOI:** 10.62641/aep.v53i3.1948

**Published:** 2025-05-05

**Authors:** Jie Yu, Lin Zhu, Yanan Song, Biyan Shi, Xiaoping Zhou

**Affiliations:** ^1^Department of Anesthesiology, The First Affiliated Hospital of Jinzhou Medical University, 121000 Jinzhou, Liaoning, China; ^2^Department of Nursing, The First Affiliated Hospital of Jinzhou Medical University, 121000 Jinzhou, Liaoning, China; ^3^Department of Neurology, The First Affiliated Hospital of Jinzhou Medical University, 121000 Jinzhou, Liaoning, China

**Keywords:** Alzheimer’s disease, holistic nursing, psychological state, cognitive function, psychiatric symptoms

## Abstract

**Background:**

Alzheimer’s Disease (AD) affects millions of elderly individuals worldwide and has been clinically recognized as one of the most significant disorders compromising quality of life in late-stage human development. The objective of this study is to systematically evaluate the influence of holistic nursing (HN) on patients with AD, thereby providing evidence-based references for clinical practice.

**Methods:**

A total of 105 patients with AD hospitalized in our hospital between January 2023 and January 2024 were enrolled for prospective analysis. Among them, 58 received conventional care (control group), and 47 received HN (observation group). Before and following the nursing interventions, both groups underwent assessment using the Mini-Mental State Examination (MMSE), Neuropsychiatric Inventory (NPI), Alzheimer’s Disease Assessment Scale-Cognitive Subscale (ADAS-cog), Montreal Cognitive Assessment (MoCA), and Self-rating Anxiety/Depression Scales (SAS/SDS). In addition, neurotransmitter levels and neuroinflammatory markers were measured using enzyme-linked immunosorbent assay and fully automated chemiluminescent immunoassay. Treatment compliance, incidence of adverse events, and family satisfaction were also recorded and compared between the two groups.

**Results:**

After nursing interventions, the observation group demonstrated significantly higher MMSE and MoCA scores compared to the control group. Conversely, NPI, ADAS-cog, SAS, and SDS scores were notably lower in the observation group (*p* < 0.05). Furthermore, neurotransmitter levels were significantly elevated in the observation group, while the concentrations of central nervous system-specific protein β (S100β) and interleukin-1β (IL-1β) were significantly reduced (*p* < 0.05). Although the incidence of adverse events did not differ significantly between the two groups (*p* > 0.05), the observation group exhibited higher treatment compliance and greater family satisfaction (*p* < 0.05).

**Conclusion:**

HN effectively improves cognitive function and alleviates psychiatric symptoms in AD patients, supporting its recommendation for clinical application.

**Clinical Trial Registration:**

No. NCT06868004.

## Introduction

Alzheimer’s disease (AD) is a neurodegenerative disorder of the central nervous system. Characterized by insidious, progressive cognitive decline and accompanying behavioral impairments, it stands as the leading cause of dementia [1]. The onset of AD predominantly occurs beyond the fifth decade of life, with the risk of incidence increasing exponentially with advancing age [2]. At present, AD affects millions of elderly individuals globally and has been clinically identified as one of the most significant disorders compromising quality of life in late-stage human development [3]. Notably, a statistical report has indicated that in 2019, mortality due to AD reached 121,499 cases, ranking as the sixth leading cause of death in the United States [4]. Despite extensive research endeavors, the precise pathogenesis of AD remains elusive, resulting in a lack of definitive and curative treatment modalities in clinical practice. In response, an increasing body of research is focusing on optimizing clinical nursing interventions for AD patients, aiming to enhance their long-term prognosis and overall quality of life.

Holistic nursing (HN) represents a progressive paradigm in nursing practice. Distinguishing itself from conventional nursing approaches, HN necessitates that healthcare professionals consider a multifaceted array of elements, including the patient’s environment, psychological disposition, and physical conditions, which can significantly impact disease rehabilitation [5]. At its core, HN is guided by a contemporary nursing philosophy, with the nursing process as its foundation, standardizing every aspect of clinical nursing and nursing management [6]. Clinically, HN is recognized for its potential to deliver more patient-centered and personalized medical services, making it particularly appropriate for patients with chronic diseases who require long-term medical support [7]. Previous research has demonstrated that the implementation of HN can enhance health outcomes in elderly hypertensive patients [8] and mitigate the incidence of complications among cancer patients undergoing chemotherapy [9]. These findings suggest that HN may similarly contribute to improving the prognosis of patients with AD. However, no direct evidence has yet been reported to confirm this hypothesis.

Therefore, the present study aimed to systematically evaluate the impact of HN on AD patients, thereby determining its clinical value in AD management. The findings are expected to provide novel references and guidelines for future rehabilitation strategies in AD care.

## Materials and Methods

### Research Subjects

We prospectively enrolled AD patients at First Affiliated Hospital of Jinzhou Medical University between January 2023 and January 2024 and randomly assigned them to receive either conventional care or HN for analysis. Patients receiving HN were designated as the observation group, while those receiving conventional care formed the control group. The required sample size for this study was calculated using G-power 3.1 software (Gamma Technologies, San Diego, CA, USA). The parameter settings were as follows: test family = *t*-test, type of power analysis = a priori, tail = two-sided, effect size = 0.5, α = 0.05, power = 0.95. The results indicated that a minimum of 42 study subjects in each group were required. Inclusion criteria were as follows: a confirmed diagnosis of AD; receipt of either conventional care or HN at our facility; age between 60 and 80 years; mild-to-moderate disease severity; adequate self-care ability and cognitive function sufficient to complete all questionnaires; and availability of complete medical records.

**Fig. 1.**
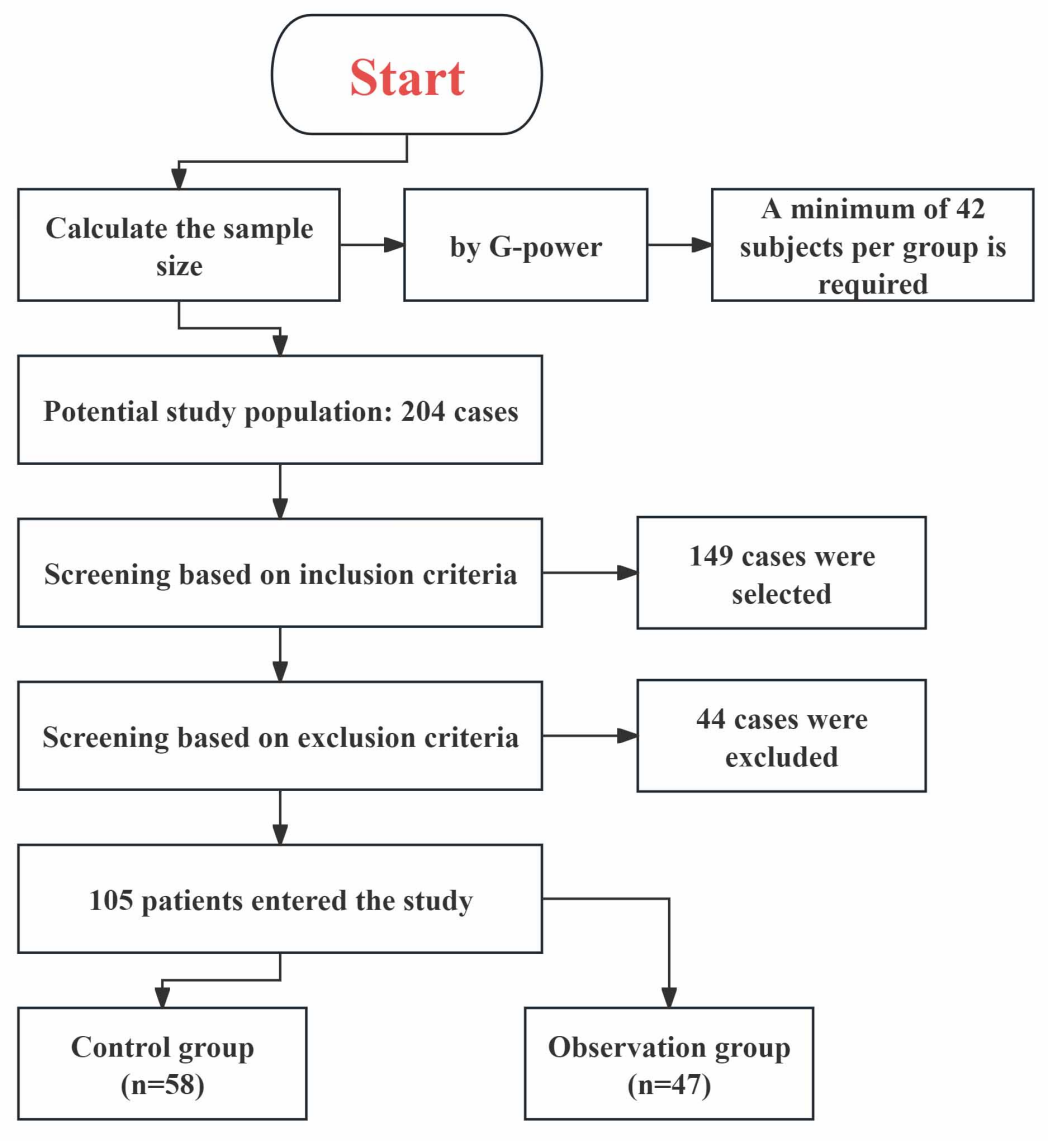
**Flowchart of the screening process for research participants**.

A stringent exclusion process was subsequently implemented. Patients with a history of intracranial surgery, pre-existing mental illness or severe psychiatric manifestations, significant impairments in vision, hearing, language, or limb motor function, dysfunction of vital organs, coexisting autoimmune disorders, or severe malignancies were excluded. Finally, 47 patients were included in the observation group and 58 in the control group. Fig. 1 illustrates the process for screening research subjects.

The study was approved by the Ethics Committee of the First Affiliated Hospital of Jinzhou Medical University (approval No. 2023199), and clinical trial registration has been completed (No. NCT06868004, https://clinicaltrials.gov/study/NCT06868004?cond=NCT06868004&rank=1). All study participants signed informed consent forms. This study was conducted in accordance with the principles outlined in the Declaration of Helsinki.

### Methods

All patients were hospitalized for seven days, during which the same nursing team provided care throughout the hospitalization period. Upon admission, all patients were administered Donepezil Hydrochloride Tablets (Guizhou Shengjitang Pharmaceutical Co., Ltd., Approval Number: H20040751, Guiyang, China) at a dosage of 10 mg once daily.

Conventional nursing: Medical staff conducted daily ward rounds. Following the physician’s instructions, the nursing staff implemented a comprehensive set of nursing interventions, including providing patients with disease-specific education, maintaining a quiet ward environment with optimal temperature and humidity, and closely monitoring patients’ vital signs and other relevant physiological parameters. Any abnormalities were promptly reported to the attending physicians.

Holistic nursing (HN) was administered with reference to HN practices for older adults with cognitive impairment described by Walker VG and Walker EK [10]: (1) Psychological intervention: Nursing staff first conducted in-depth assessments of patients’ psychological states. Based on each patient’s psychological condition, tailored communication strategies and methods were employed, and substantial time was dedicated to providing companionship. Continuous encouragement and consolation were given to enhance patients’ trust in the medical team. With patience, the nursing staff worked to alleviate patients’ negative emotions. Family members were also guided to adjust their psychological states and provide increased companionship and support. This collaborative approach aimed to help patients maintain a consistently positive and optimistic outlook in coping with their illness.

(2) Cognitive intervention: A series of structured activities, including painting and paper cutting, were introduced to enhance patients’ executive functions. Card and picture recognition tasks, along with block-building exercises, targeted attention, coordination, and fine motor skills. Family members were encouraged to actively participate in the cognitive rehabilitation process by telling stories to patients and facilitating in-depth analysis of the narrative content. Family members played a pivotal role in enhancing patients’ memory and comprehension abilities by posing reflective questions and guiding patients through problem-solving processes.

(3) Memory intervention: Personalized memory aids were created, featuring photographs and names of nursing staff, family members, and medical practitioners. Following detailed oral introductions, patients were guided to memorize this information and subsequently asked to recall and identify the corresponding individuals based on instructions, thereby training their memory. Additionally, family members were trained to assist patients in retrieving childhood and early life memories using photos, music, clothing, and other familiar items that could trigger past recollections.

(4) Self-care ability intervention: A daily schedule was prominently displayed in the ward. Repetitive daily-life training was employed to help patients develop good daily routines and form conditioned reflexes. Under the guidance of the nursing staff, a positive nurse-patient relationship and enhanced patient compliance were fostered. Activities such as grasping tableware and other daily-use tools were arranged to enhance hand dexterity. Methods like using clear signage and position-based exercises were adopted for training of orientation. Moreover, patients received personalized guidance in performing basic self-care tasks, including dressing, maintaining oral hygiene, and toileting.

(5) Environmental intervention: The ward environment was adjusted to feature a relaxing and warm color scheme, with green plants strategically arranged. Given the patients’ limited mobility and reduced self-care abilities, careful attention was given during ward decoration to eliminate or minimize the presence of sharp edges or protruding corners. This precautionary measure was implemented to mitigate the potential risk of injury and ensure the highest safety standards within the nursing environment.

### Questionnaire Surveys

The Mini-Mental State Examination (MMSE) [11], Neuropsychiatric Inventory (NPI) [12], Alzheimer’s Disease Assessment Scale-Cognitive Subscale (ADAS-cog) [13], Montreal Cognitive Assessment (MoCA) [14], and Self-rating Anxiety/Depression Scale (SAS/SDS) [15] were administered to patients before and after the nursing interventions. The MMSE (0–30 points) assesses orientation (10 points), immediate memory (3 points), attention and calculation (5 points), delayed recall (3 points), and language and manipulation (9 points). Scores of 27–30 indicate cognitive normality, 21–26 points indicate mild cognitive impairment (MCI), 10–20 points indicate moderate dementia, and 0–9 points indicate severe dementia.

The NPI (0–48 points) evaluates 12 neuropsychiatric symptoms, with higher scores reflecting more severe symptoms. A score of 0 indicates no symptoms, 1–12 points indicate mild symptoms, 13–36 points indicate moderate symptoms, and scores more than 36 points indicate severe symptoms. The ADAS-cog (0–70 points) includes assessment of word recall (10 points), naming objects (5 points), following instructions (5 points), structural exercises (5 points), intentional exercises (5 points), orientation (8 points), word recognition (12 points), recalling instructions (5 points), verbal fluency (5 points), word-finding difficulty (5 points), and attention (5 points). Scores of 0–10 reflect normal or very mild impairment, 11–20 points suggest mild Alzheimer’s disease (AD), 21–50 points correspond to moderate AD, and scores exceeding 50 points indicate severe AD.

The MoCA (0–30 points) evaluates spatial and executive functioning (5 points), naming (3 points), attention (6 points), language (3 points), abstraction (2 points), delayed recall (5 points), and orientation (6 points). Scores of ≥26 points indicate cognitive normality (adjusted for educational level), 18–25 points suggest mild cognitive impairment (MCI), 10–17 points indicate moderate dementia, and scores below 10 points indicate severe dementia. The SAS/SDS (0–100 points) surveys each consist of 20 items rated on a 4-point scale. The raw total score is multiplied by 1.25 (rounded) to obtain the final score. SAS scores <50 indicate no anxiety, while SDS scores <53 indicate no depression.

### Blood Sample Collection and Detection

Prior to and following the nursing intervention period, 4 mL of fasting venous blood was collected from each patient. The samples were centrifuged to separate plasma, which was subsequently utilized for the determination of dopamine (DA) (E-EL-0046), acetylcholine (Ach) (E-EL-0081), 5-hydroxytryptamine (5-HT) (E-EL-0033), and γ-aminobutyric acid (GABA) (E-BC-K852-M) via enzyme-linked immunosorbent assay (ELISA). All ELISA kits were purchased from Elabscience (Wuhan, China). Additionally, levels of central nervous system-specific protein β (S100β), homocysteine (Hcy), and interleukin-1β (IL-1β) were measured using a fully automated chemiluminescence immunoassay analyzer (CL-6000i, Mindray, Shenzhen, China).

### Assessment of Treatment Compliance, Safety, and Family Satisfaction

Upon patients’ discharge, treatment compliance was assessed using the Morisky Medication Adherence Scale-8 (MMAS-8) [16]. A score of less than 6 indicated poor compliance, a score of 7 denoted moderate compliance, and a score of 8 signified good compliance. The overall compliance rate was calculated as = (number of patients with good + moderate compliance)/total number of patients × 100%. The incidence of adverse events (including falls, medication errors, wandering off, etc.) from admission to discharge was also documented. Furthermore, an anonymous nursing satisfaction questionnaire was distributed to patients’ family members. Responses were categorized as very satisfied, basically satisfied, or dissatisfied. The overall satisfaction rate was calculated as = (number of very satisfied + basically satisfied family members)/total number of family members × 100%.

### Statistical Analysis

Statistical analysis was performed using SPSS 24.0 software (IBM, Armonk, NY, USA). All figures were generated using GraphPad Prism 8 software (GraphPad Software, San Diego, CA, USA). Corrections for multiple comparisons were applied for multiple hypothesis testing (such as comparisons across multiple groups, time points, or variables). The Bonferroni correction was used to control the family-wise error rate (FWER) by adjusting the significance level to α/mα/m, where mm is the number of comparisons. For exploratory analyses involving a large number of comparisons (e.g., >10), the Benjamini-Hochberg procedure was applied to control the false discovery rate (FDR). Qualitative data were expressed as frequency and percentages [n (%)], with comparisons performed using the chi-square test. Post-hoc pairwise comparisons following significant chi-square results were adjusted using the Bonferroni correction. For quantitative data, the Shapiro-Wilk test was employed to assess distribution. Normally distributed data were expressed as mean ± standard deviation ($\bar{x}$ ± s). Between-group comparisons were performed using the independent-samples *t*-test, while within-group comparisons used the paired *t*-test. Results were expressed as [median (interquartile range)] for data not following a normal distribution, and between-group and within-group comparisons were conducted using the Mann-Whitney U test and the Wilcoxon rank-sum test, respectively. Adjusted *p*-values are reported where applicable and *p*-values less than 0.05 were considered statistically significant.

## Results

### No Significant Differences in Clinical Data Between the Two Groups

As this study is a prospective analysis, it was essential to first determine the comparability of the two groups of participants. Statistical analysis of baseline data, including age, gender, and disease course, revealed no statistically significant differences between the groups (*p* > 0.05; Table 1).

**Table 1.** **Baseline comparison of clinical characteristics between groups [n (%)/($\bar{x}$ ± s)]**.

Groups	Gender		Age (years)	Body mass index	Duration of disease	Level of education	
	Male [n (%)]	Female [n (%)]	($\bar{x}$ ± s)	(kg/m^2^) ($\bar{x}$ ± s)	(months) ($\bar{x}$ ± s)	≤Junior high school [n (%)]	≥High school [n (%)]
Control (n = 58)	24 (41.38)	34 (58.62)	65.76 ± 3.64	22.58 ± 1.75	19.22 ± 4.63	34 (58.62)	24 (41.38)
Observation (n = 47)	18 (38.30)	29 (61.70)	66.17 ± 2.65	22.85 ± 2.35	19.47 ± 3.39	30 (63.83)	17 (36.17)
χ^2^ (*t*)	0.103		0.649	0.679	0.301	0.296	
*p*-value	0.749		0.518	0.499	0.764	0.586	

### The Observation Group Exhibited Milder Cognitive Function Impairment and Psychiatric Symptoms Than the Control Group

**Fig. 2.**
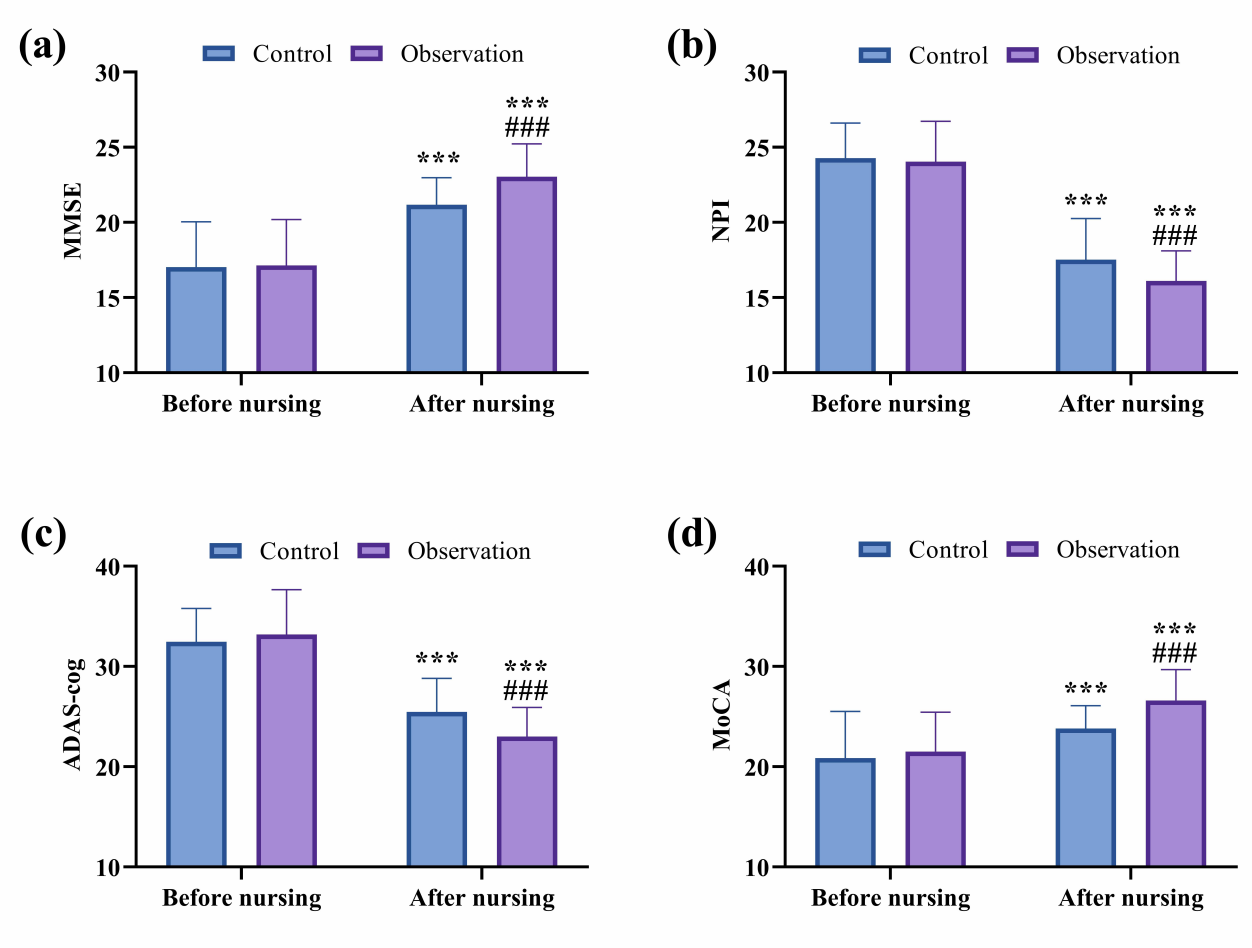
**Comparison of cognitive function and psychiatric symptoms between groups**. (a) Comparison of MMSE. (b) Comparison of NPI. (c) Comparison of ADAS-cog. (d) Comparison of MoCA. ^*⁣**^p < 0.001, comparison of post-nursing with pre-nursing data within the same group; ^#⁢#⁢#^*p* < 0.001, comparison of post-nursing data in the observation group with that in the control group. MMSE, Mini-Mental State Examination; NPI, Neuropsychiatric Inventory; ADAS-cog, Alzheimer’s Disease Assessment Scale-Cognitive Subscale; MoCA, Montreal Cognitive Assessment.

The MMSE, NPI, ADAS-cog, and MoCA scales were employed to evaluate the cognitive and psychiatric symptoms of patients. Inter-group comparisons showed no statistically significant differences in these four scale scores before nursing intervention (*p* > 0.05). After nursing, both groups demonstrated increases in MMSE and MoCA scores, with significantly greater improvements in the observation group compared to the control group (*p* < 0.001). Conversely, NPI and ADAS-cog scores decreased in both groups, with significantly lower scores observed in the observation group (*p* < 0.001; Fig. 2).

### The Observation Group Showed a Superior Psychological State Than the Control Group

**Fig. 3.**
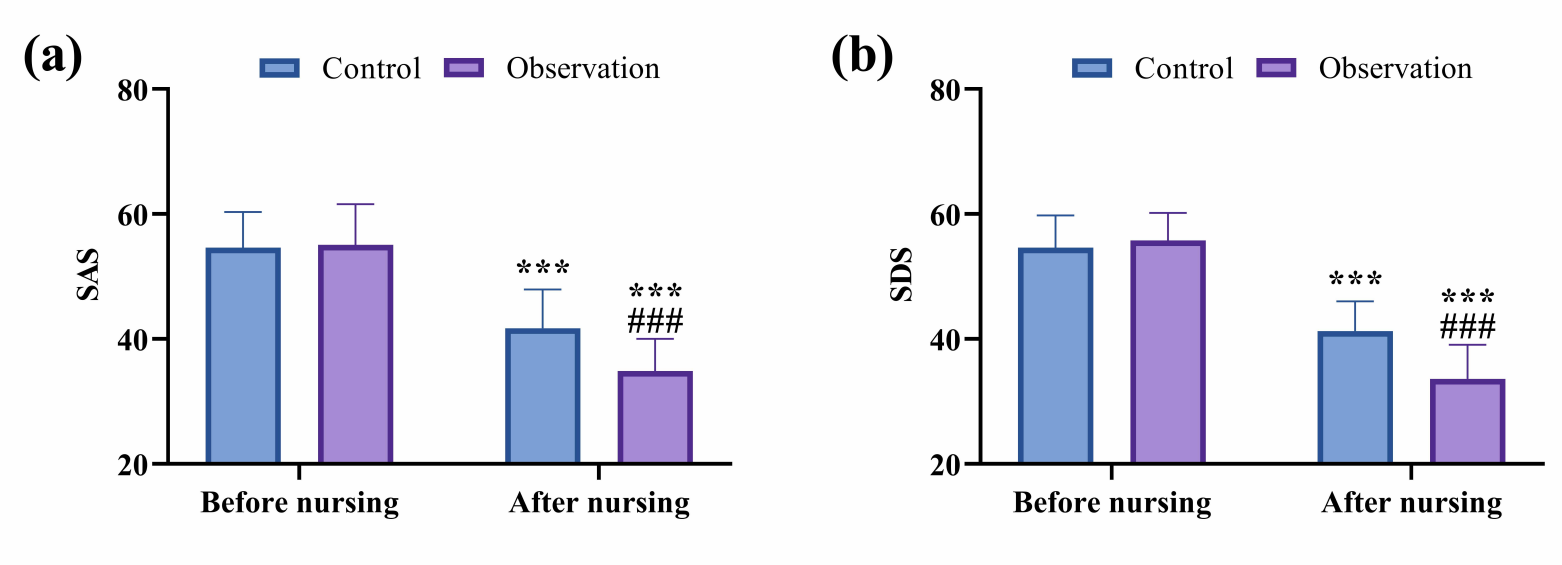
**Comparison of psychological state between groups**. (a) Comparison of SAS. (b) Comparison of SDS. ^*⁣**^p < 0.001, comparison of post-nursing data with pre-nursing data within the same group; ^#⁢#⁢#^*p* < 0.001, comparison of post-nursing data in the observation group with that in the control group. SAS/SDS, Self-rating Anxiety/Depression Scale.

Before nursing interventions, there were no significant differences in SAS and SDS scores between the two groups (*p* > 0.05). After nursing, both groups exhibited reductions in SAS and SDS scores, with significantly lower scores in the observation group compared to the control group (*p* < 0.001; Fig. 3).

### Higher Neurotransmitter Levels Were Observed in the Observation Group Compared to the Control Group

**Fig. 4.**
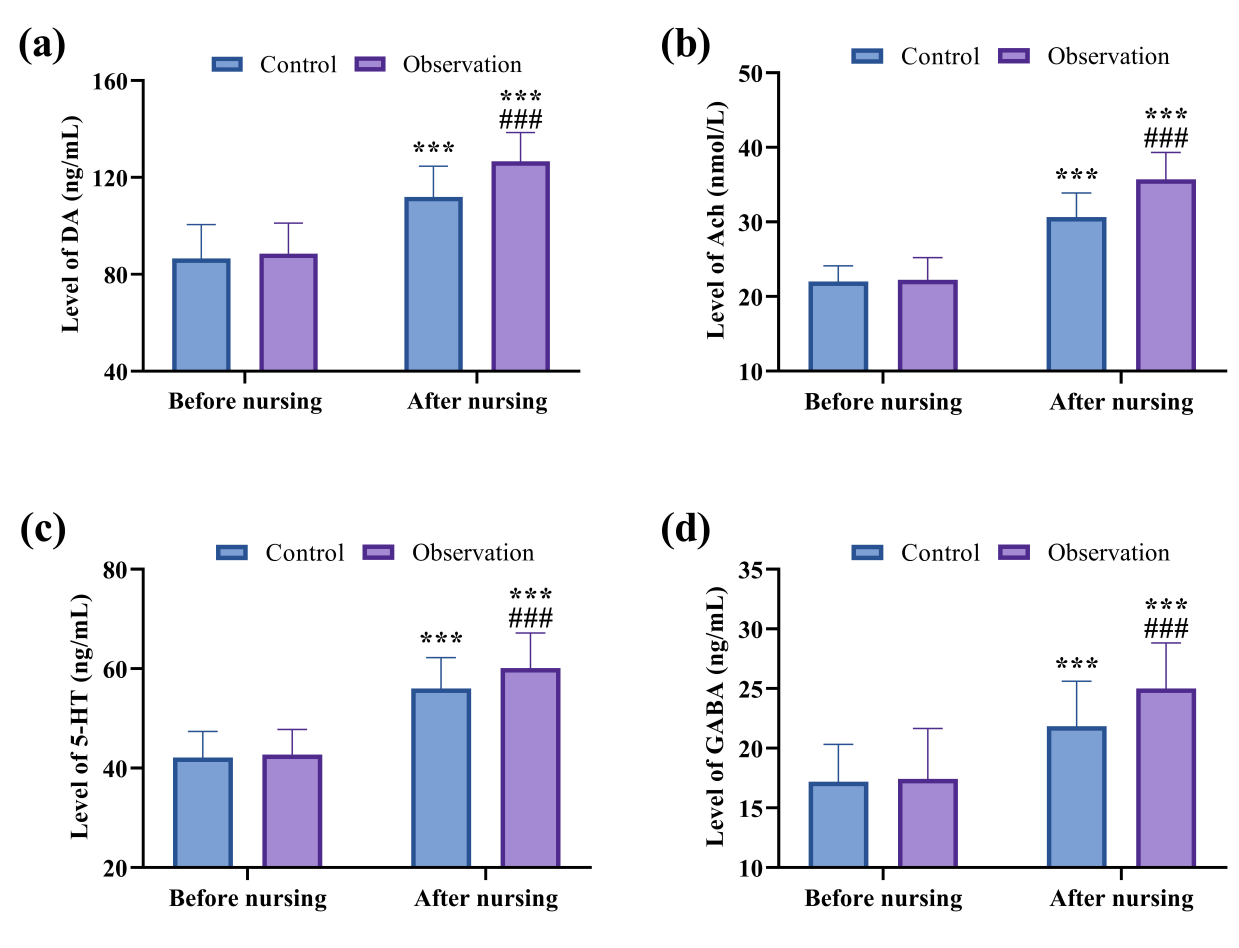
**Comparison of neurotransmitter levels between groups**. (a) Comparison of DA. (b) Comparison of Ach. (c) Comparison of 5-HT. (d) Comparison of GABA. ^*⁣**^p < 0.001, comparison of post-nursing data with pre-nursing data within the same group; ^#⁢#⁢#^*p* < 0.001, comparison of post-nursing data in the observation group with that in the control group. DA, Dopamine; Ach, acetylcholine; 5-HT, 5-hydroxytryptamine; GABA, γ-aminobutyric acid.

No significant differences were detected in pre-nursing levels of neurotransmitters (DA, Ach, 5-HT, and GABA) between the two groups (*p* > 0.05). After nursing interventions, the levels of DA, Ach, 5-HT, and GABA were significantly higher in the observation group than in the control group (*p* < 0.001). In both groups, neurotransmitter levels increased after nursing compared to pre-nursing values (*p* < 0.001; Fig. 4).

### The Observation Group Exhibited a Lower Neuroinflammatory Response Compared to the Control Group

**Fig. 5.**
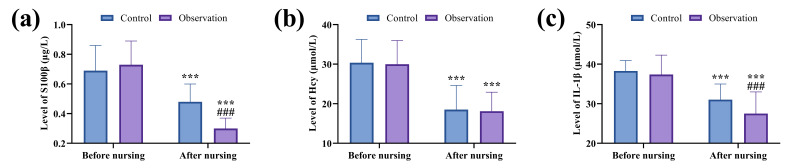
**Comparison of neuroinflammatory markers between groups**. (a) Comparison of S100β. (b) Comparison of Hcy. (c) Comparison of IL-1β. ^*⁣**^p < 0.001, comparison of post-nursing data with pre-nursing data within the same group; ^#⁢#⁢#^*p* < 0.001, comparison of post-nursing data in the observation group with that in the control group. S100β, Central nervous system-specific protein β; Hcy, homocysteine; IL-1β, interleukin-1β.

Before nursing interventions, no statistically significant differences were noted in serum levels of S100β, Hcy, and IL-1β between the two groups (*p* > 0.05). After nursing, reductions in the levels of these biomarkers were observed in both groups. Notably, S100β and IL-1β levels were significantly lower in the observation group than in the control group (*p* < 0.001; Fig. 5).

### The Observation Group Demonstrated Enhanced Treatment Compliance Than the Control Group

In the assessment of treatment compliance, the overall compliance rate in the observation group was 91.49%, representing a significant improvement compared to the control group (*p* = 0.022; Table 2).

**Table 2.** **Comparison of treatment adherence between groups [n (%)]**.

Group	Good [n (%)]	Moderate [n (%)]	Poor [n (%)]	Overall compliance rate (%)
Control (n = 58)	13 (22.41)	30 (51.72)	15 (25.86)	74.14
Observation (n = 47)	15 (31.91)	28 (59.57)	4 (8.51)	91.49
χ^2^				5.274
*p*-value				0.022

### No Significant Difference in Safety Profile Between the Two Groups

Safety assessments indicated that the incidence of adverse events in the observation group was 14.89%, compared to 24.14% in the control group. The difference between the groups was not statistically significant between the two groups (*p* > 0.05; Table 3).

**Table 3.** **Comparison of adverse events between the two groups [n (%)]**.

Group	Wrong drinking [n (%)]	Wrong diet [n (%)]	Medication errors [n (%)]	Wandering off [n (%)]	Falls [n (%)]	Abdominal pain/diarrhea [n (%)]	Other complications [n (%)]	Adverse event rate (%)
Control (n = 58)	2 (3.45)	3 (5.17)	2 (3.45)	1 (1.72)	1 (1.72)	3 (5.17)	2 (3.45)	24.14
Observation (n = 47)	1 (2.13)	2 (4.26)	0 (0.00)	0 (0.00)	1 (2.13)	2 (4.26)	1 (2.13)	14.89
χ^2^								1.387
*p*-value								0.239

### The Family Satisfaction Was Higher in the Observation Group Than in the Control Group

The nursing satisfaction survey results reported by family members indicated an overall satisfaction rate of 95.74% in the observation group, which was significantly higher than that in the control group (*p* < 0.05; Table 4).

**Table 4.** **Comparison of family satisfaction between groups [n (%)]**.

Group	Very satisfied [n (%)]	Basically satisfied [n (%)]	Dissatisfied [n (%)]	Overall satisfaction rate (%)
Control (n = 58)	20 (34.48)	28 (48.28)	10 (17.24)	82.76
Observation (n = 47)	28 (59.57)	17 (36.17)	2 (4.26)	95.74
χ^2^				4.325
*p*-value				0.038

## Discussion

Alzheimer’s disease (AD), a prevalent disorder among the elderly in contemporary society, underscores the necessity of optimizing clinical healthcare services to enhance patient prognosis. In this study, we observed that the application of HN was associated with the recovery status of AD patients.

First, based on assessments using the MMSE, NPI, ADAS-cog, and MoCA scales, we observed that the scores of MMSE, NPI, ADAS-cog and MoCA were significantly higher in the observation group than in the control group after nursing, which indicated better cognitive functioning and less severe psychiatric disorders in the observation group after care. The study by Dai J *et al*. [17] further corroborated the efficacy of HN in enhancing physiological and neurological function in jaundiced neonates, consistent with our findings. Rooted in an innovative medical model, HN scientifically integrates patients’ physiological, psychological, and social experiences, emphasizing the fulfillment of patients’ diverse needs across multiple levels, beginning with the satisfaction of basic physiological needs [18]. We hypothesize that HN enables healthcare providers to more comprehensively understand patients’ conditions. Through collaborative efforts in patient education on disease-related knowledge, HN fosters greater patient compliance with nursing interventions. Additionally, by closely monitoring psychological fluctuations and jointly providing psychological support, HN strengthens the emotional well-being of patients and instills a greater sense of security [19].

Moreover, through coordinated medical and nursing efforts in guiding patients through cognitive function training, including repetitive, systematic memory and orientation exercises, the decline of patients’ cognitive and memory functions can be decelerated. This approach promotes the reconstitution of cerebral neural function, contributing to improved cognitive function [20]. From a biological perspective, we propose that the optimization of cognitive function observed with HN in AD patients may be attributed to the following mechanisms: (1) Continuous companionship and emotional support may reduce the elevated cortisol levels induced by chronic stress, thereby reducing hippocampal neuronal damage. Improving patients’ psychological security may enhance synaptic plasticity and neuronal survival by activating the brain-derived neurotrophic factor (BDNF) signaling pathway. (2) Drawing and block-building activities activate the dorsolateral prefrontal cortex (DLPFC) and parietal cortex, which promote synaptic remodeling in brain regions associated with executive functions. Coordination exercises (e.g., paper cutting) may optimize the efficiency of interregional information transfer by increasing myelination in white matter fiber bundles such as the corpus callosum. (3) Memory recall and recollection training may stimulate the proliferation of neural stem cells in the dentate gyrus of the hippocampus, partially counteracting the decline in neurogenesis associated with AD. (4) Regular motor training (e.g., grasping and dressing) reduces movement initiation difficulties in AD patients by strengthening procedural memory and habit formation through striatal dopamine release.

Our focus on DA, Ach, 5-HT, and GABA levels is based on their well-established roles in mediating cognitive, emotional, and behavioral processes targeted by HN interventions. Central to reward processing, motivation, and motor coordination, dopamine (DA) pathways (including mesocortical and nigrostriatal pathways) are modulated through engagement in goal-directed activities (e.g., cognitive training and motor skill exercises) [21]. As a key regulator of attention, learning, and memory, acetylcholine (Ach) depletion in the basal forebrain is recognized as a hallmark of Alzheimer’s pathology [22]. Additionally, 5-HT modulates emotional stability and stress resilience [23]. GABA, the primary inhibitory neurotransmitter in the brain, maintains excitatory-inhibitory balance within the cerebral cortex [24]. These are supported by the elevated levels of DA, Ach, 5-HT, and GABA observed in the observation group after nursing, as observed in the neurotransmitter comparison between the two groups.

Moreover, by intensifying the guidance on patients’ activities of daily living through strategies such as on-site demonstrations and positive reinforcement, patients’ self-care abilities in daily life can be effectively enhanced. This alleviates the caregiving burden on families and society and enhances the overall quality of life of patients. Similarly, in terms of neuroinflammatory response, levels of S100β and IL-1β in the observation group were significantly lower following nursing interventions. Research has established that S100β, a glial-derived protein secreted by neural cells, exhibits a positive correlation with the severity of brain tissue injury [25]. Disruption of the blood-brain barrier in AD allows large amounts of S100β to enter the bloodstream [26]. IL-1β, on the other hand, is intricately involved in the inflammatory cascade of AD. It mediates the activation and proliferation of T and B lymphocytes, enhances natural killer (NK) cell activity, and induces the release of multiple inflammatory mediators [27]. These findings suggest that the safe, low-stress environment provided by HN reduces microglial overactivation, decreases pro-inflammatory cytokine release, and delays neuroinflammation-induced neuronal injury.

On the other hand, emerging evidence has demonstrated a significant association between the onset of AD and patients’ psychological well-being. A favorable psychological state may potentially reduce the incidence of AD [28]. Under the intervention of HN, medical staff, in collaboration with the patient’s family members and guided by the principles of humanism, provide nursing care akin to that of family members. They treat patients as their kin, offering understanding and care in all circumstances. By serving patients with enthusiasm and sincerity and by supporting family members in their compassionate care of patients, individuals feel respected, supported, and understood. This, in turn, strengthens the emotional connection between nurses and patients and alleviates patients’ anxiety and depressive symptoms. Consequently, in this study, the SAS and SDS scores in the observation group after nursing interventions were lower than those in the control group. Notably, in a nursing study on advanced gastric cancer, Wen Y *et al*. [29] similarly reported that the application of HN helped mitigate patients’ negative emotions, corroborating the findings of this study.

In terms of treatment compliance and patient satisfaction, the performance of the observation group was significantly superior to that of the control group. In the study by Jaramillo M [30], it was noted that HN could enhance family involvement. Therefore, we postulate that this outcome can be attributed to HN actively involving patients’ family members in the caregiving process. It allows them to understand the purpose and significance of the nursing interventions, thereby securing their support and cooperation. Additionally, providing training to family members enhances their understanding of the theoretical and practical skills of the nursing plan. This, in turn, contributes to improving the overall quality of patient care and reducing the risk of adverse events. Moreover, positive emotional experiences during the intervention may create a comfortable environment for AD patients, fostering feelings of pleasure and comfort, which is conducive to improving their compliance with intervention protocols and increasing nursing satisfaction. A study by Ji Y and Yang Y [31] on the application of HN in patients with acute myocardial infarction also supports this perspective. However, there was no significant difference in the incidence of adverse events between the two groups in the present study.

Nevertheless, based on the findings of the aforementioned studies, we expected that the incidence of adverse events in the observation group would be lower than that in the control group. We hypothesize that this discrepancy may be due to random variation resulting from the small sample size. Therefore, it is necessary to increase the number of cases in future studies to further verify the effect of HN on the safety of AD patients. In subsequent research, we should increase the sample size and conduct randomized controlled trials to further validate the clinical application value of HN in AD management. Additionally, due to the short follow-up period, we were unable to assess the long-term prognostic impact of HN on AD patients at this stage. Thus, a long-term follow-up of the study participants is warranted to evaluate the sustained prognostic implications of HN in AD care. Finally, although prospective analyses can largely reduce patient case selection bias, we still cannot fully account for the influence of certain confounding factors (e.g., patient condition, family’s level of medical care, etc.). Therefore, it is essential to conduct randomized controlled trials as soon as possible to improve the reliability and generalizability of the study findings.

## Conclusion 

HN has demonstrated significant efficacy in ameliorating the cognitive function and psychiatric symptoms of AD patients while also alleviating their negative emotional states. Consequently, it is recommended that the application of HN be further popularized in future clinical management of AD, providing a more reliable safeguard for patients’ long-term prognostic health.

## Availability of Data and Materials

The data used and/or analyzed during the current study are available from the corresponding author.
